# MEX3A Modulates PPARγ Pathway Activity and Colorectal Cancer Growth

**DOI:** 10.1016/j.jcmgh.2026.101771

**Published:** 2026-03-23

**Authors:** Ana R. Silva, Alexandre Coelho, Vanessa Machado, Morgana Russel, Dalila Mexieiro, Ana L. Amaral, Bruno Cavadas, Nuno Mendes, Carina Carvalho-Maia, Davide Gigliano, Carmen Jerónimo, Raquel Almeida, Bruno Pereira

**Affiliations:** 1 i3S – Instituto de Investigação e Inovação em Saúde, Universidade do Porto, Porto, Portugal; 2 FMUP – Faculty of Medicine, University of Porto, Porto, Portugal; 3 ICBAS – School of Medicine and Biomedical Sciences, University of Porto, Porto, Portugal; 4 HEMS – Histology and Electron Microscopy Scientific Platform, i3S, Universidade do Porto, Porto, Portugal; 5 Cancer Biology and Epigenetics Group, IPO Porto Research Center (CI-IPOP), Portuguese Oncology Institute of Porto (IPO Porto) and Porto Comprehensive Cancer Center (P.CCC) Raquel Seruca/CI-IPOP@RISE (Health Research Network), Porto, Portugal; 6 Department of Pathology, Portuguese Oncology Institute of Porto (IPO Porto), Porto, Portugal; 7 IPATIMUP – Institute of Molecular Pathology and Immunology, University of Porto, Porto, Portugal; 8 FCUP – Biology Department, Faculty of Sciences, University of Porto, Porto, Portugal

**Keywords:** Colorectal Cancer, Mouse Models, Patient-Derived Tumoroids, RNA

## Abstract

**Background & Aims:**

RNA-binding proteins (RBPs) are major effectors of post-transcriptional regulation. Recently, we described the role of Mex-3 RNA binding family member A (MEX3A) in maintaining leucine-rich repeat-containing G-protein coupled receptor 5 (LGR5)+ intestinal stem cells identity and epithelial renewal. This work aimed to study MEX3A functional impact in colorectal cancer (CRC).

**Methods:**

We characterized MEX3A expression profile in CRC mouse models and a cohort of CRC cases (n = 172). Mouse CRC tissues were used for the establishment of tumoroids and CRISPR/Cas9-mediated MEX3A knockout was performed in patient-derived CRC tumoroids to further understand its biological and therapeutic relevance. Simultaneously, we implemented the high-throughput technique HyperTRIBE to uncover MEX3A RNA targets.

**Results:**

Intestinal adenomas from *Apc*^*+/fl*^ mice have increased *Mex3a* expression, and *Apc*^*+/fl*^;*Mex3a*^*+/-*^ animals presented a significant reduction in tumor burden. *Apc*^*+/fl*^;*Kras*^*+/G12D*^;*Mex3a*^*+/-*^ compound mice exhibited reduced tumor area, whereas corresponding tumoroids had reduced growth ability and enhanced differentiation potential associated with increased peroxisome proliferator-activated receptor gamma (PPARγ) signaling. MEX3A overexpression was observed in 85% of human CRC cases, whereas 72% presented PPARγ downregulation, with a significant inverse correlation (*P* = .039). Accordingly, MEX3A-depleted patient-derived CRC tumoroids showed decreased *LGR5* expression, accompanied by increased PPARγ expression and higher sensitivity to 5-fluorouracil/oxaliplatin (FOLFOX)-based chemotherapy. HyperTRIBE results revealed a direct interaction between MEX3A and *PPARG* transcripts.

**Conclusions:**

MEX3A contributes to colorectal carcinogenesis, in association with PPARγ signaling modulation, impacting tumor development and therapeutic response.

## Graphical abstract

**Figure:**
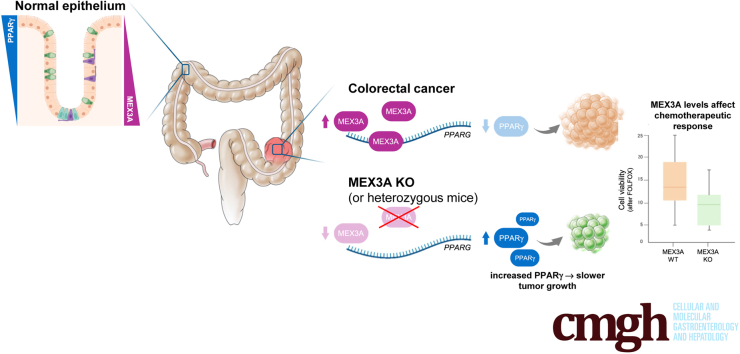

**Summary**

*Mex3a* is overexpressed in colorectal tumors. Its loss reduces tumor burden in murine models and enhances chemotherapy sensitivity in patient-derived tumoroids, in association with increased peroxisome proliferator-activated receptor gamma expression. HyperTRIBE identifies *PPARG* messenger RNA as a direct MEX3A target.

**What You Need to Know**

**Background**

Mex-3 RNA binding family member A (MEX3A) is an RNA-binding protein required for leucine-rich repeat-containing G-protein coupled receptor 5 (LGR5)+ intestinal stem cell identity and epithelial renewal by repressing the peroxisome proliferator-activated receptor gamma (PPARγ) differentiation pathway. However, MEX3A specific function and impact in colorectal cancer (CRC) are poorly characterized.

**Impact**

This study shows MEX3A loss suppresses tumor growth in mouse CRC models and increases chemotherapy sensitivity in patient-derived tumoroids. By defining the first comprehensive MEX3A interactome in CRC, we uncover *PPARG* as one of its novel RNA targets.

**Future Directions**

Future work should evaluate the therapeutic potential of targeting MEX3A in CRC, both to impair tumor progression and to enhance the efficacy of standard chemotherapy regimens.

RNA-binding proteins (RBPs) are the main mediators of the post-transcriptional regulatory machinery and are involved in virtually every RNA molecule-related step, including processing, localization, stability, translation, and turnover.^1^^,^^2^ Together with noncoding RNAs, they form ribonucleoprotein complexes that coordinate an additional and reversible layer of protein regulation.^3^^,^^4^ RBPs recognize specific structural features and/or motifs in transcripts through well-defined RNA-binding domains, such as K homology (KH) domains.^5^ Disturbances of their fine-tune functions underlie several pathological conditions,^6^ and there is growing evidence that tumor cells highjack RBP-mediated processes with repercussions in cancer hallmarks.^7^^,^^8^ Colorectal cancer (CRC) remains the third most prevalent cancer worldwide and the second cause of cancer-related deaths. Hence, the description of new biomarkers with prognostic value or amenable to targeted therapies is extremely important.

The Mex-3 RNA binding family member A (MEX3A) belongs to the evolutionary-conserved MEX-3 family of RBPs.^9^ In vertebrates, the family has 4 homologous genes (*MEX3A* to *MEX3D*), encoding proteins that are 95% identical between human and mouse.^10^ These possess 2 KH domains that mediate RNA target binding, and a really interesting new gene (RING) finger domain with E3 ubiquitin ligase activity.^11^^,^^12^ MEX3A colocalizes with the bona-fide intestinal stem cell (ISC) marker leucine-rich repeat-containing G-protein coupled receptor 5 (LGR5) during intestinal homeostasis.^13^^,^^14^ Through the characterization of the first *Mex3a* knockout (KO) mouse model, this protein was established as a functionally relevant ISC marker.^15^ *Mex3a* KO mice present retarded growth and a postnatal lethality phenotype, with a striking loss of the *Lgr5+* ISC pool, accompanied by upregulation of the peroxisome proliferator-activated receptor gamma (PPARγ) pathway.^15^ Studies have shown that PPARγ activation causes differentiation of cancer cell lines, including those derived from colon.^16^ On the other hand, MEX3A has been shown to be overexpressed in several cancer types, including breast,^17^ brain,^12^ and lung.^18^ Recently, it was proposed that drug-tolerant persister CRC cells, which mediate relapse after chemotherapy, also express MEX3A.^19^ However, the functional impact of MEX3A in the colorectal carcinogenic process has not yet been systematically addressed, nor its RNA targets.

Through in-depth characterization of CRC mouse models, human CRCs, and ex vivo tumoroid cultures, we provide evidence that MEX3A contributes to colorectal carcinogenesis. MEX3A was overexpressed in early stages of the carcinogenic cascade, detected in murine *Apc* mutant colonic adenomas and *Apc*;*Kras* mutant colon adenocarcinomas. Importantly, its downregulation led to a significantly reduced tumor burden in both mouse models. Mouse-derived tumoroids with decreased *Mex3a* expression exhibited morphological and molecular changes reminiscent of a more differentiated phenotype, associated with increased PPARγ signaling and replicated upon treatment with a PPARγ agonist. In agreement with this, MEX3A expression was substantially increased in a cohort of patients with stage II colon cancer and inversely correlated with PPARγ levels. Additionally, patient-derived tumoroids with CRISPR/Cas9-mediated MEX3A depletion had decreased *LGR5* messenger RNA (mRNA) levels accompanied by increased PPARγ protein expression, and higher sensitivity to chemotherapy. Finally, by employing the HyperTRIBE (Targets of RNA-binding proteins Identified By Editing) methodology, we defined a list of putative MEX3A RNA targets, including a direct interaction with *PPARG* transcripts. Taken together, these findings show that MEX3A is relevant for CRC development and therapeutic outcome in association with PPARγ pathway modulation.

## Results

### Increased *Mex3a* Expression and Loss of PPARγ Are Early Molecular Events During Colorectal Carcinogenesis

To characterize MEX3A expression during colorectal carcinogenesis and examine its association with the most common signaling pathways involved in this process, we first characterized the *Mex3a* mRNA expression profile in mouse models with *Apc* inactivation, *Kras* activation, and both mutations combined (n = 5). Animals carrying the corresponding *loxP* alleles were crossed with *Fabpl*^*+/cre*^ mice to generate deletions specifically in the distal small intestine and colonic epithelia.

**Figure 1:**
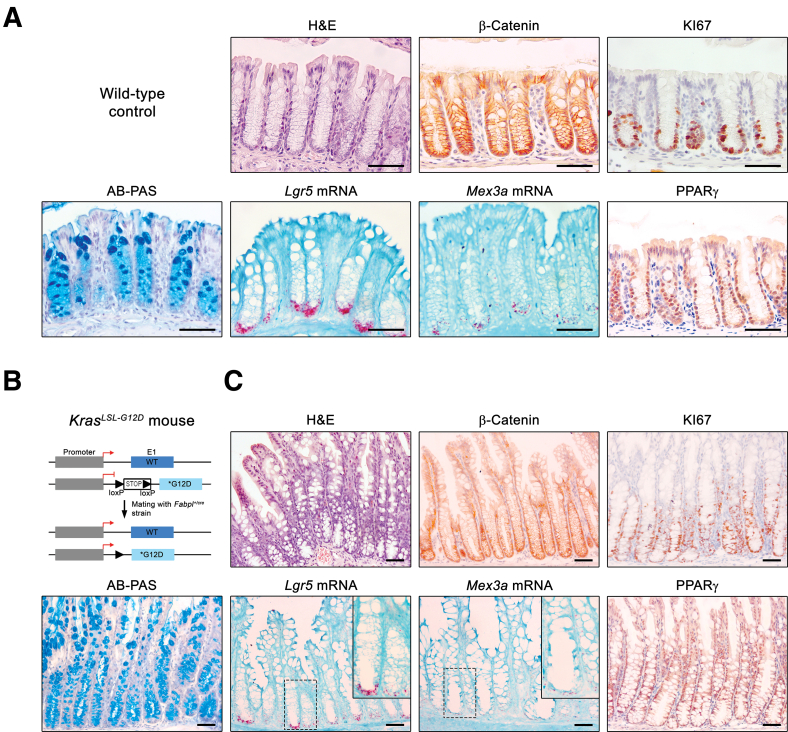
***Mex3a* and *Lgr5* mRNA expression in WT and *Kras*^*+/G12D*^;*Fabpl*^*+/cre*^ mice.** (*A*) Representative histology of colonic mucosa from WT adult mice (H&E and AB-PAS staining; β-catenin, KI67 and PPARγ IHC; *Lgr5* and *Mex3a* mRNA ISH). Scale bars, 50 μm. (*B*) Schematic representation of the genetically modified *Kras* locus for conditional induction of a constitutively active *Kras*^*G12D*^ mutant gene upon Cre-mediated recombination under the control of the *Fabpl* gene promoter. (*C*) Representative histopathology of *Kras*^*+/G12D*^;*Fabpl*^*+/Cre*^ colonic mucosa (H&E and AB-PAS staining; β-catenin, KI67 and PPARγ IHC; *Lgr5* and *Mex3a* mRNA ISH). Inserts depict high magnification of the boxed areas. Scale bars, 100 μm.

**Figure 2:**
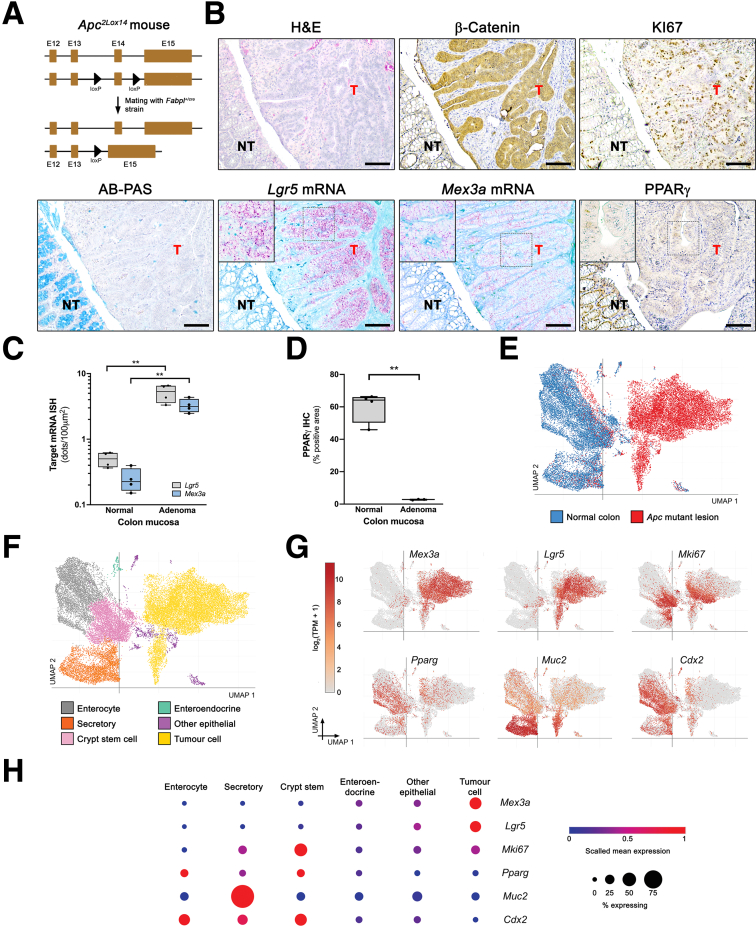
***Apc* mutant mice colon adenomas show increased *Mex3a* levels and downregulated PPARγ expression.** (*A*) Representation of the genetically modified *Apc* locus for conditional inactivation by Cre recombinase. (*B*) Histopathology of *Apc*^*+/fl*^;*Fabpl*^*+/cre*^ mice adenomas (H&E and AB-PAS staining; β-catenin, KI67 and PPARγ IHC; *Lgr5* and *Mex3a* mRNA ISH), identifying areas of tumor (T) formation and adjacent normal-like tissue (NT). Inserts depict high magnification of boxed areas. Scale bars, 100 μm. (*C*) Quantification of *Mex3a* and *Lgr5* mRNA expression levels (number of *red dots*) in T and NT normalized to 100 μm^2^ of tissue area. Data are represented in a box-and-whisker plot as mean (*middle line*) with the minimum and maximum distribution values. Each point depicts tissue areas from independent mice (n = 4). Adenoma vs normal tissue: *Lgr5* mRNA, ∗∗*P* = .0048; *Mex3a* mRNA, ∗∗*P* = .0022; unpaired Student’s *t*-test with Welch’s correction. (*D*) Quantification of PPARγ protein expression level (percentage of positive area). Data are represented in a box-and-whisker plot as mean (*middle line*) with the minimum and maximum distribution values. Each point depicts tissue areas from independent mice (n = 4). Adenoma vs normal tissue: ∗∗*P* = .0012; unpaired Student’s *t*-test with Welch’s correction. (*E*) Single-cell transcriptomic profiling of normal colon and *Apc*^*fl/fl*^;*Villin*^*creERT2*^ mice adenomas (single-cell portal). UMAP embedding of epithelial cells colored by tissue origin, comparing normal colon epithelial cells (n = 13,303) and *Apc* mutant cancer cells (n = 14,892). (*F*) UMAP embedding of the same cells colored by transcriptionally defined cell type, including enterocytes (n = 5434), secretory cells (n = 3552), crypt stem cells (n = 4739), enteroendocrine cells (n = 146), other epithelial cell types (n = 783), and tumor cells (n = 13,541). (*G*) Feature plots showing the expression level [log_2_(TPM + 1)] of marker genes across the UMAP space, highlighting stem-like (*Mex3a*, *Lgr5*), proliferative (*Mki67*), and differentiated (*Pparg*, *Muc2*, *Cdx2*) cell subpopulations. (*H*) Dot plot summarizing the expression of the same marker genes (rows) across annotated cell types (columns). Dot size represents the fraction of cells expressing each gene within each cluster, and dot color indicates scaled mean expression level.

Histological analysis of the colonic mucosa of wild-type (WT) adult mice mucosa revealed the anticipated delimitation between the differentiated and proliferative compartments, with the former comprising the top two-thirds of the crypts, as evidenced by the presence of alcian blue (AB)+ mature goblet cells, and the latter comprising the bottom third of the crypt, as indicated by KI67 staining (Figure 1*A*). *Mex3a* transcripts were restricted to the bottom of the crypt, localizing within the spatial domain of *Lgr5*+ cells (Figure 1*A*). In contrast, PPARγ showed a broader expression pattern extending towards the upper regions of the colonic crypt (Figure 1*A*). The *Kras*^*+/G12D*^;*Fabpl*^*+/cre*^ mice (Figure 1*B*) developed widespread hyperplasia throughout the colonic epithelium, reminiscent of human hyperplastic polyps. This was characterized by notable crypt lengthening, mainly due to an extension of the KI67+ cell pool and an increased number of prominent AB+ goblet cells (Figure 1*C*). No discernible differences in relation to the WT controls were observed concerning Wnt activity, *Lgr5*+ and *Mex3a*+ stem cells, and PPARγ expression profile (Figure 1*C*). In *Apc*^*+/fl*^;*Fabpl*^*+/cre*^ mice, stochastic loss of heterozygosity at the *Apc* locus (Figure 2*A*) led to the spontaneous development of focal adenomas. These lesions were clearly identifiable by Wnt pathway overactivation, as shown by increased expression of nuclear β-catenin, accompanied by increased proliferative capacity (ectopic KI67+ cells) and decreased cellular differentiation, as indicated by the reduction in the number of AB+ goblet cells (Figure 2*B*). A striking increase in the number of *Lgr5*+ cells was observed within these lesions (*P* = .0048), which appeared to be mainly accumulated at the base of the adenomatous glands (Figure 2*B* and *C*), a phenomenon previously described in other mouse models and human tissues.^20^^,^^21^ Concomitantly, we observed an increased number of *Mex3a* mRNA expressing cells (*P* = .0022), with a higher expression level (red spot intensity) per tumor cell when compared with the adjacent normal colonic tissue (Figure 2*B* and *C*). Previously, we reported a MEX3A-mediated regulation of PPARγ signalling critical for intestinal homeostasis.^15^ Here, we observed that PPARγ expression was strongly reduced in adenomas (*P* = .0012) (Figure 2*B* and *D*). Taking advantage of a publicly available single-cell RNA sequencing (scRNA-seq) dataset mapping pre-clinical mouse models of CRC,^22^ deposited in the Single Cell Portal (https://singlecell.broadinstitute.org/single_cell), we assessed the transcriptional profile of adenomas derived from *Apc*^*fl/fl*^;*Villin*^*creERT2*^ mice (Figure 2*E* and *F*). Cell-type annotated transcriptomics revealed an enrichment for *Lgr5* and *Mex3a* mRNA expression levels together with *Pparg* downregulation specifically in *Apc* mutant tumor cells (Figure 2*G* and *H*).

**Figure 3:**
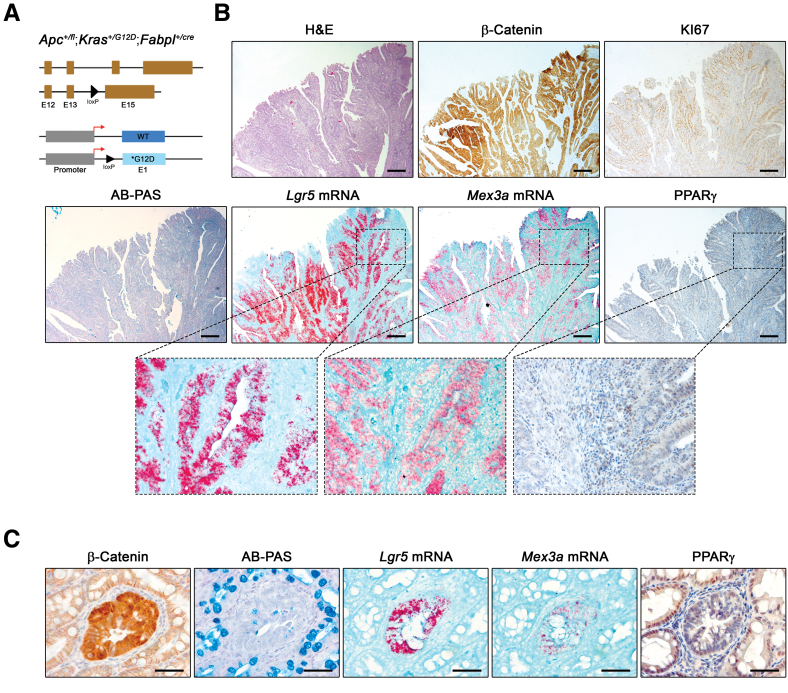
***Apc*^*+/fl*^;*Kras*^*+/G12D*^;*Fabpl*^*+/cre*^ mice colon adenocarcinomas present strong accumulation of *Mex3a*^*+*^ cells accompanied by loss of PPARγ expression.** (*A*) Representation of the modified *Apc* and *Kras* loci in the intestinal epithelium after crossing with *Fabpl*^*+/cre*^ mice. (*B*) Histopathology of *Apc*^*+/fl*^;*Kras*^*+/G12D*^;*Fabpl*^*+/cre*^ mice adenocarcinomas (H&E and AB-PAS staining; β-catenin, KI67 and PPARγ IHC; *Lgr5* and *Mex3a* mRNA ISH). Inserts depict high magnification of boxed areas. Scale bars, 200 μm. (*C*) Histopathology of a single transformed foci in *Apc*^*+/fl*^;*Kras*^*+/G12D*^;*Fabpl*^*+/cre*^ mice surrounded by normal-like tissue (AB-PAS staining; β-catenin and PPARγ IHC; *Lgr5* and *Mex3a* mRNA ISH). Scale bars, 50 μm.

When combined with *Apc* inactivation, *Kras* activation promotes the progression of adenomatous lesions to adenocarcinomas.^23^ Hence, colonic lesions in *Apc*^*+/fl*^;*Kras*^*+/G12D*^;*Fabpl*^*+/cre*^ animals (Figure 3*A*) were markedly different from those arising in the *Apc* single mutants: they developed faster (animals were euthanized around 10 weeks of age compared with 30 weeks on average for *Apc* mutant mice) and were more extensive and heterogeneous (Figure 3*B*). Strong accumulation of *Lgr5*+ and *Mex3a*+ cells was confined to tumor tissue showing high Wnt activity, as evidenced by increased β-catenin nuclear expression, together with the presence of KI67+ cells and loss of AB+ cells (Figure 3*B*). PPARγ expression was almost absent, but again only in areas with enhanced Wnt activity (Figure 3*B*). Localized transformation events were also visible, which may indicate putative crypts of origin (Figure 3*C*). These transformed foci, recognized by β-catenin increased expression and loss of AB staining, already showed ectopic *Mex3a*+ and *Lgr5*+ cells, with a concomitant decrease in PPARγ expression (Figure 3*C*). Overall, these results demonstrate coordinated molecular changes linking Wnt pathway activation, increased *Mex3a* expression, and PPARγ downregulation during CRC development.

### *Mex3a* Heterozygosity Impairs Adenoma Formation in an *Apc*-Inactivation Background

**Figure 4:**
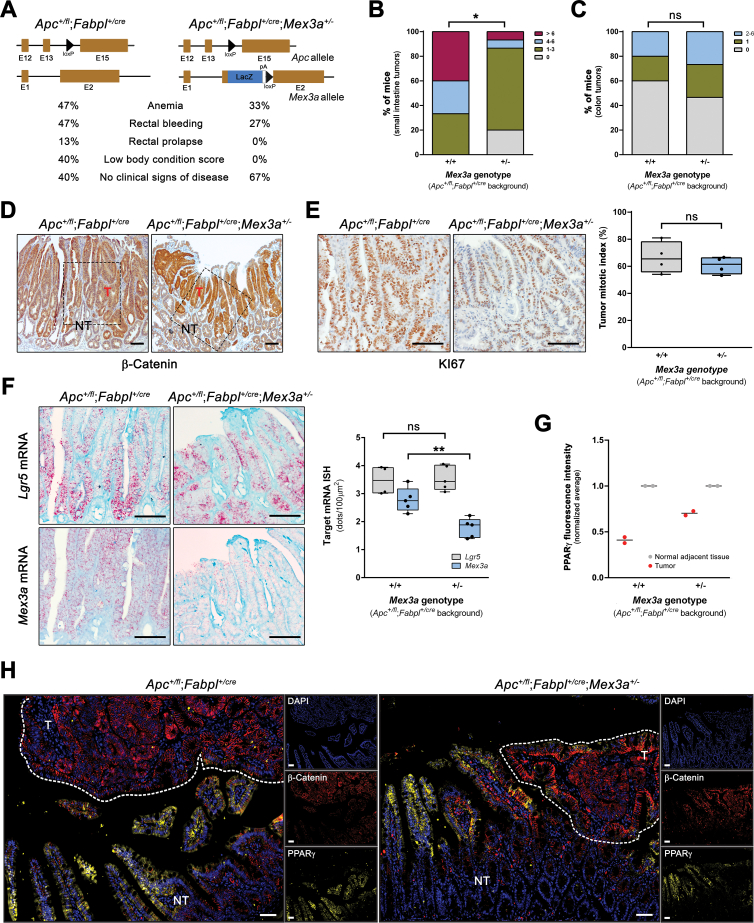
***Mex3a* downregulation impairs adenoma formation in an *Apc*-inactivation background.** (*A*) Percentage of animals with different *Mex3a* genotypes in an *Apc*^*+/fl*^;*Fabpl*^*+/cre*^ background presenting signs of disease (n = 15, each genotype). (*B*) Quantification of the number of tumors in the ileum of *Apc*^*+/fl*^;*Fabpl*^*+/cre*^ (+/+) and *Apc*^*+/fl*^;*Fabpl*^*+/cre*^;*Mex3a*^*+/-*^ (+/−) mice. *+/*− vs *+/+* adenomas: ∗*P* = .0182; χ^2^ test. (*C*) Quantification of the number of tumors in the colon of +/+ and +/− mouse models. *+/*− vs *+/+* adenomas: *P* = .7650, not significant (ns); χ^2^ test. (*D*) β-Catenin overexpression in both +/+ and +/− mice ileal lesions, identifying areas of tumor (T) formation, lined by adjacent normal-like tissue (NT). The boxed areas are shown in panels *E* and *F* stained for the indicated markers. Scale bars, 100 μm. (*E*) KI67 staining in *+/+* and *+/*− mice adenomas and tumor mitotic index (percentage of actively dividing cells per total number of cells within the tumor). Data are represented in a box-and-whisker plot as mean (*middle line*) with the minimum and maximum distribution values. Each point depicts tissue areas from independent mice (n = 4). *+/*− vs *+/+* adenomas: *P* = .411, ns; unpaired Student’s *t*-test. Scale bars, 100 μm. (*F*) *Lgr5* and *Mex3a* mRNA ISH in *+/+* and *+/*− mice adenomas. Quantification of *Mex3a* and *Lgr5* mRNA expression levels (number of *red dots*) normalized to 100 μm^2^ of tissue area. Data are represented in a box-and-whisker plot as mean (*middle line*) with the minimum and maximum distribution values. Each point depicts tissue areas from independent mice (n = 4). +/− vs +/+ adenomas: *Lgr5* mRNA, *P* = .81, ns; *Mex3a* mRNA, ∗∗*P* = .0022; unpaired Student’s *t*-test with Welch’s correction. (*G* and *H*) Multiplex immunofluorescence staining of β-catenin and PPARγ proteins in *+/+* and *+/*− tumors, with quantification of PPARγ fluorescence intensity for paired tumors and normal-like adjacent tissue. Each point depicts independent tissue areas (n = 2). Scale bars, 100 μm.

To address the role of MEX3A in adenoma initiation, independent cohorts of age-matched *Apc*^*+/fl*^;*Fabpl*^*+/cre*^;*Mex3a*^*+/-*^ and *Apc*^*+/fl*^;*Fabpl*^*+/cre*^ animals (n = 15) were obtained. We observed that *Apc*^*+/fl*^;*Fabpl*^*+/*cre^ mice exhibited signs of disease more frequently than *Apc*^*+fl*^;*Fabpl*^*+/cre*^;*Mex3a*^*+/-*^ littermates (60% vs 33%, respectively) (Figure 4*A*). The number of lesions in the small intestine and colon was quantified (Figure 4*B* and *C*), revealing a significant decrease (*P* = .018) in tumor burden in *Apc*^*+/fl*^;*Fabpl*^*+/cre*^;*Mex3a*^*+/-*^ mice, specifically in the ileum (Figure 4*B*). Although 20% (3/15) of the *Apc*^*+/fl*^;*Fabpl*^*+/cre*^;*Mex3a*^*+/-*^ animals had no lesions and 67% (10/15) showed 1 to 3 tumors in the terminal part of the small intestine, all *Apc*^*+/fl*^;*Fabpl*^*+/cre*^ mice developed lesions, with 67% (10/15) having 4 or more tumors (Figure 4*B*). These lesions showed increased expression of nuclear β-catenin (Figure 4*D*) and the absence of differentiated AB+ goblet cells (not shown), while showing a similar mitotic index (Figure 4*E*). *Lgr5* and *Mex3a* expression levels were increased when compared with adjacent normal-like tissue in both genotypes (Figure 4*F*), with *Mex3a* heterozygous animals showing a significant reduction in *Mex3a* mRNA levels when compared with *Mex3a* WT animals, as expected (*P* = .0022) (Figure 4*F*). Simultaneously, PPARγ expression was highly reduced in transformed cells of both genotypes when compared with the adjacent normal-like tissue (Figure 4*G* and *H*). On the other hand, tumor burden in the colon was low and similar for both genotypes, with about one-half of all animals not presenting lesions (Figure 4*C*). The few tumors that grew showed expression profiles of the different lineage markers similar to the ones observed in the small intestinal tumors (not shown). These data imply that MEX3A expression is not absolutely required for Wnt-mediated tumor initiation, but its reduced expression and/or function influences tumor growth.

### *Mex3a* Downregulation Reduces CRC Burden in an *Apc*^*+/fl*^;*Kras*^*+/G12D*^ Background and Alters CRC Tumoroid Growth Dynamics

**Figure 5:**
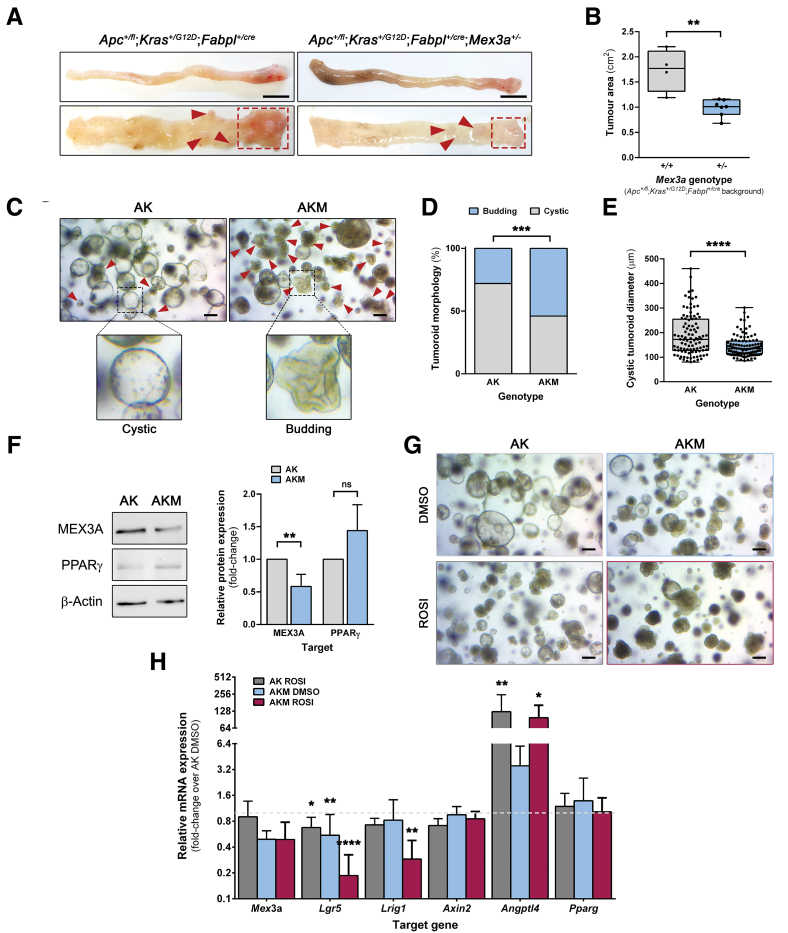
***Apc*^*+/fl*^;*Kras*^*+/G12D*^;*Fabpl*^*+/cre*^*;Mex3a*^*+/*^**^−^**mice present lower tumor burden and altered tumoroid growth dynamics.** (*A*) Macroscopic images of the colon from *Apc*^*+/fl*^;*Kras*^*+/G12D*^;*Fabpl*^*+/cre*^ and *Apc*^*+/fl*^;*Kras*^*+/G12D*^;*Fabpl*^*+/cre*^*;Mex3a*^*+/*−^ mice. *Boxed areas* and *red arrows* indicate tumor location. Scale bars, 1 cm. (*B*) Quantification of tumor area in *Apc*^*+/fl*^;*Kras*^*+/G12D*^;*Fabpl*^*+/cre*^ (+/+) and *Apc*^*+/fl*^;*Kras*^*+/G12D*^;*Fabpl*^*+/cre*^*;Mex3a*^*+/*−^ (+/−) colon (n = 4 and n = 7, respectively). Data are represented in a box-and-whisker plot as mean (*middle line*) with the minimum and maximum distribution values. +/− vs +/+ tumor area: ∗∗*P* = .0021; unpaired Student’s *t*-test. (*C*) Phase-contrast microscopy images of *Apc*^*+/fl*^;*Kras*^*+/G12D*^;*Fabpl*^*+/cre*^ (AK)- and *Apc*^*+/fl*^;*Kras*^*+/G12D*^;*Fabpl*^*+/cre*^ (AKM)-derived colon tumoroids after 7 days of culture. *Red arrows* indicate budding structures and inserts depict high magnification of boxed regions. Scale bars, 100 μm. (*D*) Quantification of the proportion of budding and cystic morphologies in AK and AKM tumoroids (n = 3). AKM vs AK group: ∗∗∗*P* = .0003; Fisher’s exact test. (*E*) Quantification of the size (diameter length) of AK and AKM cystic tumoroids (n = 3). Data are represented in a box-and-whisker plot as mean (*middle line*) with the minimum and maximum distribution values. Each point depicts one tumoroid. AKM vs AK group: ∗∗∗∗*P* < .0001; Mann-Whitney test. (*F*) Western blot of MEX3A and PPARγ proteins extracted from AK and AKM tumoroid cultures and quantification of expression levels (n = 3). Data is presented as mean fold-change plus SD. AKM vs AK group: MEX3A, ∗∗*P* = .0088; PPARγ, *P* = .063, not significant (ns); unpaired Student’s *t*-test. (*G*) Phase-contrast microscopy images of AK and AKM cultures after 5 days of treatment with 50 μM ROSI or equal volume of the drug vehicle (DMSO). Scale bars, 100 μm. (*H*) RT-qPCR analysis of *Mex3a*, *Lgr5*, *Lrig1*, *Axin2, Angptl4*, and *Pparg* mRNA expression levels in AK and AKM tumoroid cultures after ROSI treatment (n = 3). Data is presented as the mean fold-change plus SD for target gene expression relative to DMSO-treated AK tumoroids (*dashed line*). AK ROSI vs AK DMSO: *Lgr5*, ∗*P* = .02; *Angptl4*, ∗∗*P* = .002; AKM DMSO vs AK DMSO: *Lgr5*, ∗∗*P* = .0011; AKM ROSI vs AK DMSO: *Lgr5*, ∗∗∗∗*P* < .0001; *Lrig1*, ∗∗*P* = .0083; *Angptl4*, ∗*P* = .017; 1-way ANOVA with Dunnett’s correction for multiple comparisons.

Due to the development of few colonic lesions in the *Apc* mutant strains, we established independent cohorts of age-matched *Apc*^*+/fl*^;*Kras*^*+/G12D*^;*Fabpl*^*+/cre*^;*Mex3a*^*+/-*^ and *Apc*^*+/fl*^;*Kras*^*+/G12D*^*;Fabpl*^*+/cre*^ animals (n = 7 and n = 4, respectively). Macroscopic examination revealed a 43% decrease (*P* = .0021) in tumor growth area in the distal colon of *Apc*^*+/fl*^;*Kras*^*+/G12D*^;*Fabpl*^*+/cre*^;*Mex3a*^*+/-*^ mice (0.99 ± 0.17 cm^2^) when compared with *Apc*^*+/fl*^;*Kras*^*+/G12D*^;*Fabpl*^*+/cre*^ control animals (1.73 ± 0.42 cm^2^) (Figure 5*A* and *B*). Lesions were not found in the small intestine.

To directly examine MEX3A role in cancer cell behavior, we established *Apc*^*+/fl*^;*Kras*^*+/G12D*^;*Fabpl*^*+/cre*^;*Mex3a*^*+/-*^ (AKM)- and *Apc*^*+/fl*^;*Kras*^*+/G12D*^;*Fabpl*^*+/cre*^ (AK)-derived tumoroid cultures (n = 3). These were kept in culture media solely containing the growth factor noggin (NOG) for selective expansion of tumor cells. AKM cultures had a significantly higher proportion of budding tumoroids, in contrast to the predominance of cystic structures in AK cultures (*P* = .0003) (Figure 5*C*, red arrows and inserts). More than one-half of AKM tumoroids were morphologically reminiscent of normal budding intestinal organoids (Figure 5*D*), suggesting increased cell differentiation. When comparing the cyst-like structures only, AKM tumoroids were also significantly smaller than AK controls (*P* < .0001) (Figure 5*E*).

Given our previous observations concerning the *Mex3a* KO mouse model,^15^ we examined whether changes in PPARγ signaling might relate to the tumoroids phenotypic changes. To address this, we first assessed MEX3A and PPARγ protein expression in AK and AKM tumoroids. As expected, MEX3A protein expression was significantly decreased in AKM tumoroids (*P* = .0088) (Figure 5*F*). PPARγ protein abundance was generally low, mimicking the expression profile observed in tumor tissues. Still, there was a trend for increased PPARγ expression in AKM tumoroids (*P* = .063) (Figure 5*F*). Then, to establish a functional link, and given the low basal level of PPARγ expression, we treated tumoroid cultures with the PPARγ-selective agonist rosiglitazone (ROSI) for 5 days. ROSI treatment induced an overall decrease in tumoroid size alongside a striking change in tumoroid morphology, namely a reduction in the number of cystic structures, mostly noticeable in AK cultures (Figure 5*G*). Treatment efficiency was confirmed by increased *Angptl4* mRNA expression levels, a PPARγ transcriptional target, whose expression was already endogenously enhanced in AKM cultures (Figure 5*H*). Interestingly, ROSI treatment induced a marked decrease in expression levels of the *Lgr5* and *Lrig1* ISC markers, particularly in AKM tumoroids, when compared with dimethyl sulfoxide (DMSO)-treated AK tumoroids (*P* < .0001 and *P* = .0083, respectively) (Figure 5*H*). This effect was not due to Wnt signaling blockade, since the expression of the canonical Wnt target gene *Axin2* did not change (Figure 5*H*). Of note, *Pparg* mRNA expression also did not change in the different conditions. These results show that *Mex3a* downregulation decreases CRC growth in vivo and is associated with a more differentiated phenotype alongside increased PPARγ activity.

### MEX3A Is Overexpressed in Human CRC Tissues

**Figure 6:**
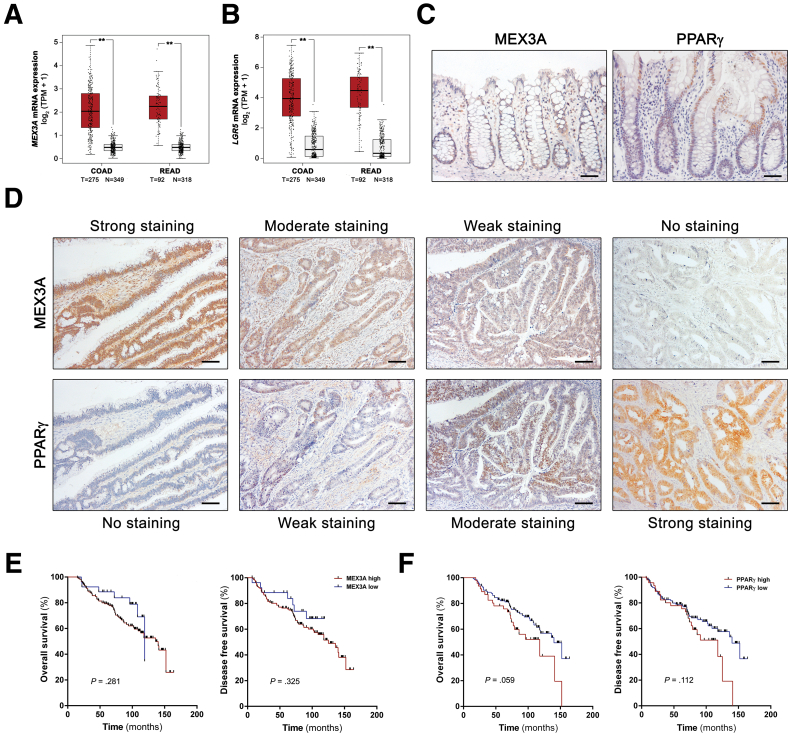
**MEX3A and PPARγ are differentially expressed in human colon tumors.** (*A* and *B*) Analysis of *MEX3A* (*A*) and *LGR5* (*B*) mRNA expression levels from TCGA and the nondisease state GTEx portal (expression in log_2_ scale). N, normal; T, tumor. T vs N: ∗∗ *P* < .01; 1-way ANOVA test. (*C*) Representative IHC for MEX3A and PPARγ in human normal colon tissues. Scale bars, 50 μm. (*D*) Representative IHC for MEX3A and PPARγ in the same cancer cases, portraying tumors with strong, moderate, weak and negative staining. Scale bars, 50 μm. (*E* and *F*) Kaplan-Meier curves of overall and disease-free survival according to MEX3A (*E*) and PPARγ (*F*) expression levels in a cohort of patients with stage II CRC. *P* values are depicted in the respective plot; log-rank (Mantel-Cox) test.

To explore the MEX3A expression profile in human CRC cases, we performed a bioinformatic analysis with data from the cancer data-mining platform The Cancer Genome Atlas (TCGA) and the nondisease state Genotype-Tissue Expression (GTEx) portal. This revealed significantly increased *MEX3A* mRNA expression levels (*P* < .01) in both colon (COAD) and rectal (READ) adenocarcinomas when compared with normal tissues (Figure 6*A*). The same significant difference was observed for *LGR5* (*P* < .01) (Figure 6*B*). To obtain insights regarding MEX3A expression and tissue distribution in early cancer stages, we analysed MEX3A protein levels by immunohistochemistry (IHC) in a cohort of patients with stage II colon cancer (n = 172). In normal colon tissue (n = 5), MEX3A staining was faintly detected at lower crypt regions (Figure 6*C*). On the other hand, in 84.9% of tumor tissues (146/172), MEX3A presented increased expression, with moderate to strong staining in over 66% of the cancer cells and mainly at the nuclear level (Figure 6*D*). In the remaining 15.1% (26/172), MEX3A showed a weak staining pattern, with few negative cases. The clinicopathological data for the cohort and association with the MEX3A expression profile are summarized in Table 1. High MEX3A expression was significantly associated with microsatellite stable (MSS) tumors (*P* = .007). No significant associations were found for other clinicopathological parameters.

**Table 1:** Summary of Clinicopathological and Molecular Associations With MEX3A and PPARγ Expression Levels in Colon Cancer

	Total	MEX3A			PPARγ		
		Low	High	*P* value	Low	High	*P* value
Age, *y*							
Mean	69	67	69	.246	68	71	.117
SD	11	11	11		11	10	
Gender							
Female	71 (41.3)	7.0	34.3	.584	29.4	11.2	.908
Male	101 (58.7)	8.1	50.6		43.5	15.9	
Histological grade							
G1	3 (1.7)	0.0	1.7	.497	0.6	1.2	.147
G2	158 (91.9)	14.5	77.3		67.1	25.3	
G3	11 (6.4)	0.6	5.8		5.3	0.6	
Tumor location							
Proximal	68 (39.5)	6.5	33.7	.678	28.7	10.8	.949
Distal	101 (58.7)	8.3	51.5		43.7	16.8	
ND	3 (1.7)						
Microsatellite instability							
MSS	89 (51.7)	5.1	51.6	**.007**	32.9	23.9	**<.0001**
MSI	68 (39.5)	10.8	32.5		40.0	3.2	
ND	15 (8.7)						
*BRAF* status							
WT	139 (80.8)	12.5	70.2	.780	62.7	20.5	.478
V600E	29 (16.9)	3.0	14.3		11.4	5.4	
ND	4 (2.3)						
SOX9							
Low	16 (9.3)	3.0	6.6	.079	7.9	1.8	.316
High	151 (87.8)	12.6	77.8		64.8	25.5	
ND	5 (2.9)						
PPARγ							
Low	124 (72.1)	13.5	59.4	**.039**			
High	46 (26.1)	1.8	25.3				
ND	2 (1.2)						
MEX3A							
Low	26 (15.1)						
High	146 (84.9)						

NOTE. Results are expressed as a percentage or mean (SD).

NOTE. Boldface *P* values indicate statistical significance.

MEX3A, Mex-3 RNA binding family member A; MSI, microsatellite instable; MSS, microsatellite stable; ND, not determined; PPARγ, peroxisome proliferator-activated receptor gamma; SD, standard deviation; WT, wild-type.

Additionally, we analyzed PPARγ levels in the same series. In normal colonic tissues, PPARγ was mainly detected in upper crypt regions (Figure 6*C*). Contrasting with MEX3A, 72.1% of the tumor cases (124/172) were negative or weak for PPARγ expression, whereas 26.7% (46/172) presented moderate to strong staining (Figure 6*D*). High levels of PPARγ were also significantly associated with MSS tumors (*P* < .0001), but no statistically significant associations were observed with other parameters (Table 1). Despite the observed correlations with MSS status and possible implications for patient response to treatment, MEX3A and PPARγ expression profiles did not correlate with patient outcome (Figure 6*E* and *F*). Interestingly, we were able to establish a statistically significant correlation between high MEX3A levels and lower PPARγ expression (*P* = .039). In line with our findings in murine CRC models, the data sustains that there is also an association between increased MEX3A expression and downregulated PPARγ in early stages of human colorectal carcinogenesis.

### *MEX3A* KO in Patient-Derived CRC Tumoroids Leads to Re-Expression of PPARγ and Enhances Sensitivity to Chemotherapy

**Figure 7:**
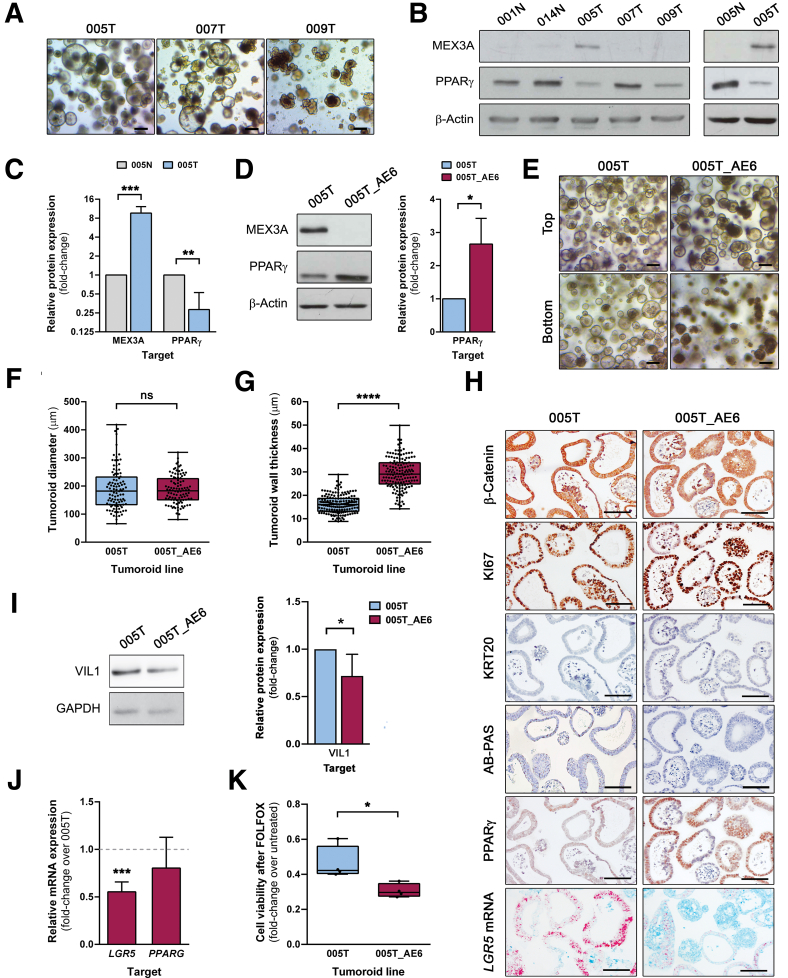
**MEX3A KO in patient-derived CRC tumoroids increases PPARγ expression and enhances chemosensitivity.** (*A*) Representative phase-contrast microscopy images of 3 PDCT lines after 14 days of culture. Scale bars, 200 μm. (*B*) Western blot of MEX3A and PPARγ expression in patient-derived CRC tumoroids and normal colon-derived organoid lines. (*C*) Quantification of MEX3A and PPARγ protein expression in 005T (tumoroids) and 005N (organoids) lines derived from the same patient (n = 4). Data is presented as mean fold-change plus SD. 005T vs 005N: PPARγ, ∗∗*P* = .001; MEX3A: ∗∗∗*P* = .0005; unpaired Student’s *t*-test. (*D*) Western blot of MEX3A and PPARγ protein expression levels in 005T_AE6 (MEX3A KO clone) and parental 005T tumoroids. Quantification of PPARγ protein expression levels in 005T_AE6 and parental 005T (n = 3). Data is presented as mean fold-change plus SD. 005T_AE6 vs 005T: ∗*P* = .0211; unpaired Student’s *t*-test. (*E*) Phase-contrast microscopy images of 005T_AE6 and 005T tumoroids at day 14 of culture, taken either near the top or the bottom of the Matrigel dome. Scale bars, 200 μm. (*F* and *G*) Quantification of the size (*F*) and wall thickness (*G*) of 005T_AE6 and 005T cystic tumoroids (n = 3). Data are represented in box-and-whisker plots as mean (*middle line*) with the minimum and maximum distribution values. Each point depicts one tumoroid. 005T_AE6 vs 005T: size, *P* = .68, not significant (ns); thickness, ∗∗∗∗*P* < .0001; Mann-Whitney test. (*H*) Histopathology of 005T_AE6 and 005T tumoroid lines at day 14 of culture (AB-PAS staining; β-catenin, KI67, KRT20 and PPARγ IHC; *LGR5* mRNA ISH). Scale bars, 100 μm. (*I*) Western blot of VIL1 protein expression levels in 005T_AE6 and parental 005T tumoroid lines and respective quantification (n = 4). Data is presented as mean fold-change plus SD. ∗*P* = .0481; unpaired Student’s *t*-test. (*J*) RT-qPCR analysis of *LGR5* and *PPARG* mRNA expression levels in 005T_AE6 and 005T tumoroid cultures after 14 days of culture (n = 6). Data is presented as the mean fold-change plus SD for target gene expression relative to 005T levels (*dashed line*). 005T_AE6 vs 005T: *Lgr5,* ∗∗∗*P* = .0001; unpaired Student’s *t*-test. (*K*) Quantification of cell viability of 005T_AE6 and 005T tumoroid lines after FOLFOX-based treatment for 72 hours (n = 4). Data are represented in a box-and-whisker plot as mean (middle line) with the minimum and maximum distribution values. 005T_AE6 vs 005T: ∗*P* = .0286; Mann-Whitney test.

To gather functional evidence of the role of MEX3A in human colon cancer cells, we established patient-derived CRC tumoroid (PDCT) lines (Figure 7*A*). In agreement with the CRC cases previously analysed, we observed an inverse correlation between MEX3A and PPARγ protein expression profiles both in PDCTs and normal colon-derived organoid lines (Figure 7*B* and *C*). The PDCT line 005T was genetically engineered using CRISPR/Cas9 technology to establish isogenic human tumoroids with a MEX3A KO (005T_AE6) (Figure 7*D*). The lack of MEX3A did not seem to significantly alter tumoroid growth kinetics in comparison to the parental line (Figure 7*E* and *F*). However, at late culture stages (around 2 weeks), MEX3A KO PDCTs had thicker walls (Figure 7*G*) and became darker in appearance, mostly the ones positioned deeper inside the Matrigel dome (Figure 7*E*). These features suggested an altered cellular differentiation profile.^24^ Most interestingly, MEX3A KO tumoroids exhibited a significant decrease in *LGR5* mRNA levels (*P* = .0001) (Figure 7*H* and *J*) accompanied by a significant increase in PPARγ protein expression (Figure 7*D* and *H*). We also tested AB-periodic acid Schiff (PAS) staining, together with KRT20 and VIL1 expression (Figure 7*H* and *I*), but did not find increased levels, suggesting the MEX3A KO tumoroids still retain some undifferentiated features.

As the MEX3A KO PDCTs characterization pointed towards lower stemness potential, we examined whether MEX3A abrogation might influence CRC cells response to chemotherapy. Thus, we exposed the MEX3A KO tumoroids and corresponding isogenic controls to a combination of 5-fluorouracil (5-FU) and oxaliplatin (FOLFOX regimen) for 72 hours. We observed that MEX3A KO tumoroids showed significantly higher sensitivity to treatment (*P* = .0286), exhibiting lower cell viability when compared with the parental line (Figure 7*K*). Collectively, these results demonstrate that MEX3A is necessary for LGR5+ stem cell subpopulation maintenance in human CRC cells, with a functional impact for chemotherapy response.

### *PPARG* mRNA Is a MEX3A Target in CRC Cells

**Figure 8:**
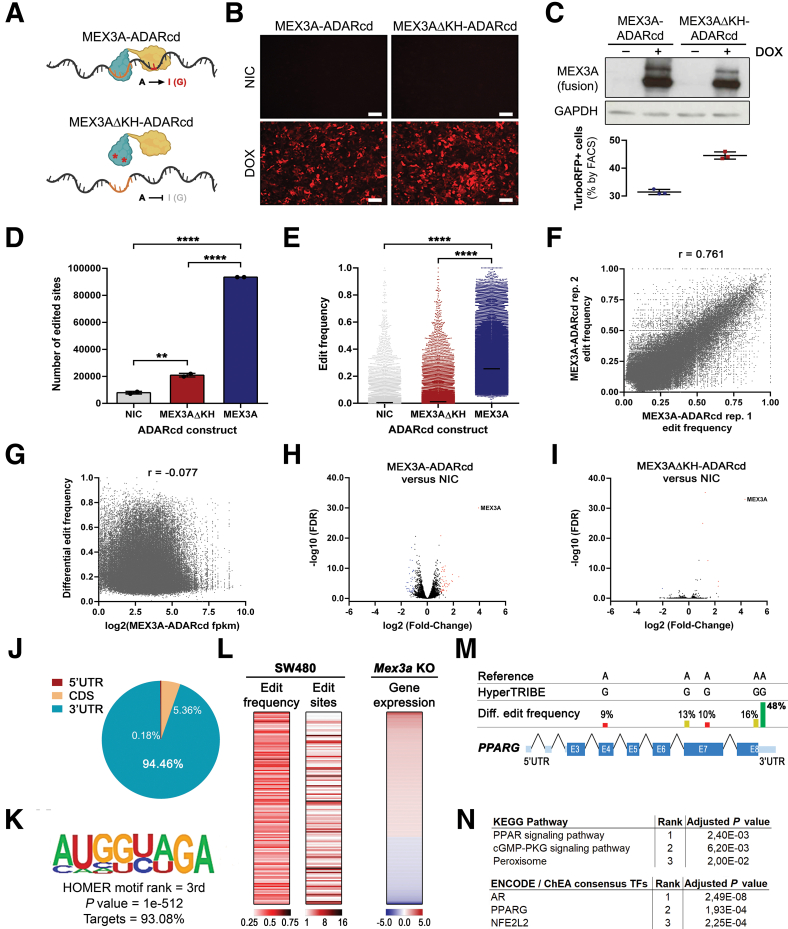
**HyperTRIBE identifies *PPARG* as a MEX3A direct target in CRC cells.** (*A*) Representation of the HyperTRIBE constructs and outputs, showing the MEX3A protein (*green*) fused to the catalytical domain of ADAR (ADARcd; *yellow*). Upon MEX3A binding to the MEX-3 recognition element (*orange motif*) in target mRNAs, ADARcd creates A-to-I RNA editing events in the vicinity (*red base*) that are identified as A-to-G mismatches in RNA sequencing. A MEX3A protein with mutations in the KH RNA-binding domains (MEX3AΔKH) serves as a control for off-target editing. (*B*) Representative images of MEX3A-ADARcd and MEX3AΔKH-ADARcd stably transduced cell lines after 48 hours of HyperTRIBE system induction with 1 μg/mL DOX and corresponding NICs. (*C*) Representative Western blot of MEX3A fusion proteins in HyperTRIBE system induction experiments in the SW480 cell line. MEX3A-ADARcd fusion proteins have a higher molecular weight (predicted around 100 kDa) than the endogenous MEX3A protein (65 kDa; not detected). Graphical representation of the average proportion of TurboRFP+ cells collected by FACS after 48h of HyperTRIBE system induction (n = 3 independent induction assays). (*D*) Number of edited sites on mRNA transcripts. Data are represented as means plus SD in 2 independent experiments. MEX3AΔKH vs NIC: ∗∗*P* = .0019; MEX3A vs MEX3AΔKH: ∗∗∗∗*P* < .0001; MEX3A vs NIC: ∗∗∗∗*P* < .0001; Tukey’s multiple comparisons test. (*E*) Edit frequency distribution of significant sites (*P* < .05). Each *dot* represents an edit event, with the mean value represented as a *black line*. MEX3A vs MEX3AΔKH: ∗∗∗∗*P* < .0001; MEX3A vs NIC: ∗∗∗∗*P* < .0001; Dunn’s multiple comparisons test. (*F*) Linear correlation analysis between the 2 independent replicates for editing frequency. r = .761. (*G*) Linear correlation analysis between editing frequency and transcript expression level (fpkm). r = −0.077. (*H* and *I*) Volcano plots of differential expressed genes in MEX3A-ADARcd (*H*) or MEX3AΔKH-ADARcd (*I*) vs NIC control. *Blue and red dots* represent genes with down or upregulated expression, respectively (*P* < .01 and −2 < fold-change > 2). (*J*) Distribution of the edit sites according to transcripts’ region. (*K*) De novo motif analysis with the HOMER software revealed an enrichment for the sequence AUGG(U/C)(A/U)GA, which overlaps with the known MRE (A/G/U)(G/U)AGN_(0–8)_U(U/A/C)UA. (*L*) Heatmap of 137 transcripts that show edit sites in SW480 cells and have significantly altered expression in *Mex3a* KO intestinal crypts. (*M*) Distribution of 5 edited sites across the *PPARG* transcript. The 3 most confident sites are the following: Exon 7 CDS: 13% edit frequency, *P* = .0015; Exon 8 CDS: 16% edit frequency, *P* = .0013; 3′UTR: 48% edit frequency, *P* = .00067. (*N*) KEGG pathway and TF analyses of the subset of 137 transcripts overlapping with the *Mex3a* KO gene expression signature. The 3 most significant terms in each analysis are depicted according to their *P* value.

In parallel with the previous functional characterization, we sought to obtain mechanistic insights about the MEX3A regulatory network. For this, we employed a cutting-edge technique called HyperTRIBE, based in the establishment of a fusion protein between the human MEX3A coding sequence (CDS) and the hyperactive version of the catalytic domain (cd) of the fruit fly RNA-editing enzyme adenosine deaminase acting on RNA (ADAR).^25^ Once expressed, it creates irreversible adenosine-to-inosine (A-to-I) RNA editing events that are identified as adenosine-to-guanosine (A-to-G) mismatches near MEX3A-binding sites (Figure 8*A*). Editing specificity is guaranteed by the lack of RNA-binding of ADARcd. To control potential off-target editing, we generated a MEX3A RNA-binding mutant (MEX3AΔKH) by introducing mutations in the KH domains hallmark GxxG loops.^26^ To prevent analytical bias by ADAR continuous activity, MEX3A-ADARcd and MEX3AΔKH-ADARcd sequences were placed under the control of a doxycycline (DOX)-inducible promoter for transient expression together with a TurboRFP reporter. Upon lentiviral transduction of the human CRC cell line SW480 and establishment of puromycin-selected cell populations, tight regulation of the system was confirmed 48 hours after DOX addition by red fluorescent protein (RFP) fluorescence detection (Figure 8*B*) and MEX3A-ADARcd fusion proteins overexpression (Figure 8*C*). RFP+ induced cells were collected by fluorescence-activated cell sorting (FACS) (Figure 8*C*) and used for RNA sequencing (RNA-seq) with the corresponding noninduced controls (NICs).

MEX3A-ADARcd protein induction in SW480 cells led to a significant increase in the absolute number of edited sites and edit frequency of RNAs when compared with NIC or overexpression of the MEX3AΔKH-ADARcd mutant (Figure 8*D* and *E*). Linear correlation analysis indicated that edit frequency was highly reproducible between replicates (Figure 8*F*) and independent of transcript expression level (Figure 8*G*) or transcript size (not shown). Transient induction (48 hours) of the MEX3A-ADARcd fusion protein did not cause extensive transcriptional changes, with only 81 transcripts significantly altered (*P* < .01 and -2 < fold-change > 2) (Figure 8*H*). Importantly, MEX3AΔKH-ADARcd protein expression essentially did not disturb the cell’s transcriptome (Figure 8*I*), confirming that the alterations observed upon MEX3A-ADARcd activation are exclusively due to MEX3A function.

To confidently pinpoint MEX3A RNA targets, we applied a set of highly stringent filtering criteria—including a 25% editing threshold, a false discovery rate (FDR) < .001, and excluding from the target list transcripts with any editing event detected in the MEX3AΔKH-ADARcd or NIC conditions—which led to the identification of 12,173 unique edited sites in 3278 transcripts (Supplementary Table 1). The majority of these sites (∼94%) were located in the 3′ untranslated region (UTR), consistent with previous findings (Figure 8*J*).^27^^,^^28^ In addition, a de novo motif search using a 1000 bp window centered at the edited sites revealed the presence of a particular sequence overlapping a known MEX-3 recognition element (MRE) within the top 3 most significantly enriched motifs^27^^,^^28^ (Figure 8*K*). We found that 137 edited transcripts were common to the *Mex3a* KO intestinal crypt gene signature,^15^ implying a direct regulatory effect (Figure 8*L*). Strikingly, *PPARG* mRNA was part of the subset of edited RNAs also upregulated upon *Mex3a* deletion, exhibiting a major edited site in the 3′ UTR (Figure 8*M*). Kyoto Encyclopedia of Genes and Genomes (KEGG) pathway and transcription factor (TF) analyses of this subset showed significant enrichment for the PPAR pathway and PPARG transcriptional targets, respectively (Figure 8*N*). Overall, the data demonstrates that MEX3A-ADARcd protein’s editing effect and regulatory function are fully dependent on MEX3A RNA-binding ability and that *PPARG* is a direct MEX3A target.

## Discussion

RBPs are crucially important for controling homeostatic cellular processes, and their altered function is associated with cancer initiation and progression. Here, using CRC mouse models, human tumor tissues, and ex vivo tumoroid cultures, we provide evidence that MEX3A plays a biologically relevant role in CRC, particularly in early stages, with its overexpression associated with LGR5+ cancer stem cell subpopulations and loss of PPARγ expression, influencing tumor development and therapeutic response.

The characterization of *Apc* mutant mice revealed a connection between this first event of malignant transformation and increased *Mex3a* levels. Although LGR5 is a target of the Wnt/β-catenin pathway,^29^ there is currently no data supporting MEX3A as a direct Wnt signaling target. As such, ectopic *Mex3a* upregulation in *Apc* mutant-derived adenomas and *Apc*;*Kras* mutant-derived adenocarcinomas presumably reflects a higher percentage of cellular subpopulations with stem cell-like features and not merely amplified β-catenin transcriptional activation. A *Mex3a*+ cell reporter was also observed to be highly expressed in colon adenomas arising from inactivation of the *Apc* locus specifically in *Lgr5*+ cells.^19^ In contrast, *Mex3a* haploinsufficiency significantly decreased CRC burden in the mouse models tested, both in terms of the number and extension of lesions. Similar tumor attenuation effects were recently reported in inflammation-induced CRC mouse models with either a constitutive^30^ or conditional^19^ *Mex3a* KO, indicating a common phenotypic outcome independent of the CRC mouse model genetic basis or the tumorigenic insult.

We noticed that MEX3A deficiency leads to differences in ex vivo tumoroid growth and morphology patterns. The alterations in AKM mouse-derived tumoroids and in MEX3A KO PDCTs were not fully identical, most probably due to their distinct mutational backgrounds. Still, the appearance of multiple organoid differentiation features,^24^^,^^31^ including frequent budding events, a darker appearance, and thick-walled cysts, all point to common changes in cancer cells indicative of a more differentiated state. In agreement, we detected lower LGR5 levels upon partial or complete MEX3A loss. Although *Mex3a* deficiency was previously associated with upregulation of a secretory-like molecular program in murine colon adenomas,^19^ we did not see increased goblet cell-like differentiation based on AB-PAS staining in both our mouse- or patient-derived models. This suggests MEX3A KO cells might become primed towards the secretory lineage without exhibiting complete functional maturation.

Most notably, the observed alterations were accompanied by increased PPARγ protein expression and signalling. On the contrary, we did not detect changes in *PPARG* mRNA expression levels (Figures 5*H* and 7*J*), which is consistent with a post-transcriptional regulation model. As a complete list of MEX3A RNA targets has not yet been determined, we performed high-throughput identification of putative MEX3A-binding transcripts by HyperTRIBE, providing the very first MEX3A RNA interactome in CRC cells. Successful adaptation of the technique was supported by the specific enrichment of MRE-like motifs in the vicinity of edited sites in 93% of the putative targets identified. Interestingly, very few of the edited targets had an altered expression level in our experimental setup (only 68, considering *P* < .05 and −1.5 < fold-change > 1.5). This might be a direct result of the short-term induction or indicative of a major role for MEX3A in translational control. Nevertheless, the results show that the editing activity reflects MEX3A-specific binding. HyperTRIBE revealed that *PPARG* mRNA is part of the MEX3A edited transcripts’ list. Although only one *PPARG* edited site passed the very strict filtering criteria established, lowering the edit frequency to a still conservative 10% threshold^25^ revealed 2 additional high-confident sites in the coding sequence, with 13% and 16% edit frequencies (Figure 8*M*), strengthening *PPARG* transcript as a true MEX3A binding target. Other elements of the PPAR pathway also presented editing events, including glycerol kinase (*GK*), sorbin and SH3 domain containing 1 (*SORBS1*), acyl-CoA oxidase 1 (*ACOX1*), and stearoyl-CoA desaturase 5 (*SCD5*). This is reminiscent of the post-transcriptional regulon model,^32^ whereby MEX3A might control the expression of functionally related transcripts in a coordinated manner to achieve fine-tuning of a specific biochemical process.

Given our in vivo and ex vivo data, we propose that *Mex3a* loss is associated with increased PPARγ expression and signaling, alongside a differentiation shift that contributes to the reduced tumor growth observed across our mouse models. In this framework, *Mex3a* deficient tumors with robust PPARγ re-expression would be less likely to develop, and indeed, we did not identify such lesions. Instead, the tumors that still arise in AKM mice likely represent “escaper” cells that fail to fully activate the PPARγ-mediated differentiation program, consistent with the low PPARγ protein levels detected both in vivo and in the derived tumoroids. Given this inherently low PPARγ protein expression, we functionally tested our hypothesis using a pharmacological activation approach. Treatment with the PPARγ-selective agonist ROSI replicated the morphological and molecular features of *Mex3a* deficiency, including a more budding-like morphology, increased *Angptl4* expression, and decreased *Lgr5* expression, thus supporting the PPARγ pathway relevance to these phenotypic changes.

PPARγ is a member of the nuclear hormone receptor superfamily, identified as a master transcriptional determinant of adipogenesis and lipid metabolism.^33^ It has since been demonstrated to regulate cellular differentiation in various biological settings, including suppression of osteoblast differentiation from mesenchymal stem cells,^34^ promotion of osteoclast differentiation from hematopoietic stem cells,^35^ and differentiation of bladder epithelial cells,^36^ as well as macrophage differentiation from monocytes.^37^ In the gut, PPARγ is primarily localized in the differentiated epithelial compartment (Figures 1 and 6*C*).^38^ On the other hand, altered PPARγ activity has been causally associated with disease, including neurological disorders, chronic inflammation, and cancer.^39^ Several in vitro studies have demonstrated that PPARγ activation causes cell cycle arrest and differentiation of cancer cell lines, including those derived from lung,^40^ prostate,^41^ breast,^42^ pancreas^43^ and colon.^44^ Available in vivo data also supports a role for PPARγ as a tumor suppressor, with *Pparg* intestine-specific KO animals showing increased tumor incidence in the context of transgenic, chemically-mediated, or inflammation-induced CRC.^45^, ^46^, ^47^ Moreover, a large cohort study suggests that PPARγ expression is independently associated with good prognosis in CRC.^48^ A complex and reciprocally opposing crosstalk exists between the canonical Wnt pathway and PPARγ signaling. However, in our CRC models, MEX3A abrogation led to increased PPARγ protein expression even in a background of Wnt/β-catenin pathway constitutive activation. This suggests that fine-tuning of PPARγ activity is particularly dependent on MEX3A and at least partially uncoupled from Wnt signaling status.

The characterization of a cohort of patients with stage II colon cancer demonstrated an inverse correlation between MEX3A and PPARγ expression levels at early CRC stages. We were also able to establish a statistically significant association between high MEX3A levels and MSS status, which might indicate a prognostic value for MEX3A, because stage II CRC patients with MSS tumors have a worse prognosis when compared with highly immunogenic microsatellite instable (MSI) tumors.^49^^,^^50^ Although we could not establish a correlation between MEX3A levels and patient’s survival, probably due to the low number of relapses in our cohort, several studies have been published in the past couple of years linking MEX3A overexpression with worse prognosis in multiple cancer types, including brain,^12^^,^^51^ breast,^17^^,^^52^ lung,^18^ liver,^53^ pancreatic,^54^ kidney,^55^ and colon.^30^ Conversely, we established the first CRC patient-derived tumoroid model with a full MEX3A deletion, showing a heightened chemotherapeutic response to FOLFOX. This is in line with a recent observation in an experimental model of CRC liver metastasis, in which triple-mutant (*Apc*^*−/−*^;*Kras*^*+/G12D*^;*Trp53*^*−/−*^) mouse-derived tumoroids with a *Mex3a*-KO also show enhanced response to a combination of 5-FU and SN-38 (FOLFIRI regimen), with significantly delayed metastatic outgrowth.^19^ An association between partial MEX3A inhibition and improved chemotherapy results has equally been reported, namely for glioblastoma cells treated with temozolomide,^51^ pancreatic ductal adenocarcinoma cell lines upon gemcitabine treatment,^54^ and hepatocellular carcinoma cell lines exposed to sorafenib,^53^ thus strengthening the concept that MEX3A downregulation generates a tumor cell vulnerability that can be therapeutically exploited in multiple cancer types.

We previously observed that overactivation of the PPARγ pathway dramatically impairs normal intestinal organoid growth and expansion due to loss of *Lgr5*+ ISCs, particularly in the absence of MEX3A.^15^ In the CRC context, despite a significant reduction in *LGR5* levels, both ROSI-treated AKM tumoroids and MEX3A KO PDCTs retained self-renewal ability. This suggests that LGR5- cancer cell subpopulations with stem-like properties might still contribute to tumor propagation upon MEX3A depletion. Our future studies will address MEX3A effect over additional signalling pathways, highly represented within the set of MEX3A-ADARcd edited transcripts, and how the MEX3A-mediated regulatory network further contributes to colorectal carcinogenesis.

## Materials and Methods

### Animal Models

Animal experimentation was performed in accordance with the Portuguese National Regulation established by Decreto-Lei 113/2013 that is the national transposition of the European Directive 2010/63/EU for the Care and Use of Laboratory Animals. Mice were bred at the i3S Animal Facility, accredited by the Association for Assessment and Accreditation of Laboratory Animal Care (AAALAC). All strains were in a C57BL/6 background. The *Mex3a* KO strain was generated at the National Centre of Biotechnology (CNB-CSIC) and previously characterized.^15^ The *Apc*^*2Lox14*^, *Kras*^*LSL-G12D*^, and *Fabpl*^*cre*^ strains (a kind gift from Sérgia Velho, i3S) were also previously established and characterized.^23^ The *Apc*^*2Lox14*^, *Kras*^*LSL-G12D*^ and *Fabpl*^*cre*^ strains were mated with the *Mex3a* heterozygous strain to obtain *Apc*^*+/fl*^*;Fabpl*^*+/cre*^*;Mex3a*^*+/-*^ (n = 15; 8 males and 7 females) and *Apc*^*+/fl*^;*Kras*^*+/G12D*^;*Fabpl*^*+/cre*^*;Mex3a*^*+/-*^ (n = 7; 5 males and 2 females) cohorts and the corresponding *Apc*^*+/fl*^*;Fabpl*^*+/cre*^ (n = 15; 8 males and 7 females) and *Apc*^*+/fl*^;*Kras*^*+/G12D*^;*Fabpl*^*+/cre*^ (n = 4; 2 males and 2 females) controls. Animals were genotyped using specific primer pairs (Table 2). Mixed cohorts were kept under a standard 12-hour light/dark cycle, with water and rodent chow available ad libitum and housed in type II polycarbonate cages in single-sex groups of 2 to 6 animals. Each cage was provided with corncob bedding (LBS Serving Biotechnology), sheets of absorbent paper, a cardboard tube (LBS Serving Biotechnology) for nesting, and an acrylic tunnel for handling. To determine the number of animals to be used for tumor development evaluation in different genetic backgrounds we used the G∗Power 3.1.9 software to perform statistical power analyses. Tumor burden (including number and/or size of lesions) was established as the primary outcome measure. Animal weights were monitored 3 times a week, and humane endpoints for euthanasia were established, including the assessment of the following parameters starting at 7 weeks of age: anemia, rectal bleeding, rectal prolapse development, and low body condition score. Upon clinical presentation of any of these indicators by one of the animals in the cage (regardless of genotype), the whole cohort was sacrificed. No sex-specific differences were observed in tumor burden or tumoroid phenotypes, and data were pooled for analysis. The researchers involved in executing the procedures are certified in animal experimentation (FELASA C).

**Table 2:** List of Primers Used in This Study

	Strand	Sequence (5′–3′)
Genotyping		
*Apc*^*2Lox14*^	Sense	GATGGGTCTGTAGTCTGGG
	Antisense	GGCTCAGCGTTTTCCTAATG
*Fabpl*^*cre*^	Sense	CCTGATCCTGGCAATTTCG
	Antisense	GGACTCACTAATGTTTGCTG
*Kras*^*LSL-G12D*^	Sense (G12D)	GTCTTTCCCCAGCACAGTGC
	Sense (WT)	AGCTAGCCACCATGGCTTGAGTAAGTCTGCA
	Antisense	CTCTTGCCTACGCCACCAGCTC
*Mex3a*_Wt	Sense	TGCAGGGTTTCTCTAAACTGG
	Antisense	ACCAGGGACATGGAGCTTAG
*Mex3a*_LacZ	Sense	ATCCTCTGCATGGTCAGGTC
	Antisense	CGTTACGCGTTCGCTCATC
mRNA expression		
*Angptl4*	Sense	ATCTTCAGAGCCAGATAGACC
	Antisense	AGCTGGGTCATCTTGGGAAG
*Axin2*	Sense	TGGCCAGTCAGCAGAGGGACA
	Antisense	TTTGGCTCTTTGTGATCTTCTGG
*Lgr5*	Sense	AGCGTCTTCACCTCCTACCTG
	Antisense	CTTGGGAATGTGTGTCAAAGC
*LGR5*	Sense	CAGCGTCTTCACCTCCTACCTA
	Antisense	CCTTGGGAATGTATGTCAGAGC
*Lrig1*	Sense	CAAGCTGACTCTGTTTGGAAAC
	Antisense	ACTGGACAGACCTGATTGC
*Mex3a*	Sense	TCTACAAAGAGGCCGAGCTG
	Antisense	GCCTTAATCTTGCAGCCTTGC
*Pparg*	Sense	CGGACAAATCACCATTTGTCATC
	Antisense	GATGGCCACCTCTTTGCTCT
*PPARG*	Sense	CAGACAAATCACCATTCGTTATC
	Antisense	GATGGCCACCTCTTTGCTCT
*18S*	Sense	CGCCGCTAGAGGTGAAATTC
	Antisense	CATTCTTGGCAAATGCTTTCG
MEX3A KO sgRNA construct assembly and sequencing (uppercase indicates gene-specific primer)		
sgRNA	Sense	caccgCCTACATCAAGACACCGGTG
	Antisense	aaacCACCGGTGTCTTGATGTAGGc
U6	Sense	gagggcctatttcccatgattcc
MEX3A KO clones sequencing		
*MEX3A*	Sense	AGAAAGGAGTGTAGTTTGTGAATGG
	Antisense	GGAAGTCGTTTTCATTGTTGTACTC
HyperTRIBE assembly (uppercase indicates gene-specific primer)		
ADARcd_CDS	Sense	aatattctccggttcaggatatTCACCAATGGTGGTGCCAC
	Antisense	aaggcgcaaccccaaccccggatcctcaTTCGGCAAGACCGAACTC
*MEX3A_CDS*	Sense	aaccccggtcctaggctgcagATGCCTAGTCTAGTGGTATC
	Antisense	ggtgaatatcctgaaccGGAGAATATTCGGATGGC
Site-directed mutagenesis (uppercase indicates gene-specific primer)		
ADARcd_hyperactive	Sense	CGAGTCCGGTcaGGGGACGATTC
	Antisense	ATTTTGGTGCGCAGCTGG
MEX3A_KH1	Sense	GATCGTGGGCgacgatGGCTGCAAGATTAAGGCCTTGAGGG
	Antisense	TCGGCCACGTGCTCGGAG
MEX3A_KH2	Sense	GGTGGTGGGCgacgatGGGGCAACCATCAAGCG
	Antisense	AGCCCCACCACGCGGTAG
HyperTRIBE plasmid sequencing		
MEX3A	Sense	CAAGCTCTGCGCTCTCTACAAA
	Antisense	GGCCTTAATCTTGCAGCCTTG
pCW57	Sense	GGCAAGATCCTCGAGTACAACA
	Antisense	ACGCCTGGAGACTATTTCAGC
	Sense	CGCGTCGAAAGGTATTAGCG
	Antisense	AAGAATGTGCGAGACCCAGG
T7 promoter	Antisense	TAATACGACTCACTATAGGG

### Samples From Patients With CRC

Formalin-fixed paraffin-embedded (FFPE) CRC tissue specimens and normal colonic tissues were obtained from the FFPE tumor tissue biobank of Centro Hospitalar Universitário de S. João (CHUSJ). Samples were collected between January 2002 and December 2010 and their use for research purposes approved by the CHUSJ Ethics Committee. The cohort included 71 female and 101 male patients (n = 172). Sex was not associated with MEX3A or PPARγ expression patterns (Table 1), and analyses were not stratified by sex. The establishment of patient-derived organoids was conducted in collaboration with the biobank of IPO Porto and approved by the IPO Ethics Committee (CES IPO: 81/022). A cohort of treatment-naïve adult patients (over 50 years old, irrespective of sex) was recruited between May 2022 and August 2023. Written informed consent was obtained from all patients prior to enrollment.

### Histochemical Procedures

FFPE mouse tissue sections with 5-μm (in situ hybridization [ISH]) or 3-μm (other procedures) thickness were deparaffinized and rehydrated following standard protocols. For morphological analysis, slides were stained with 7211 hematoxylin for 1 minute, differentiated in 1% ammoniacal water for 30 seconds, and counterstained using eosin solution for 3 minutes. For the AB-PAS staining, tissues were first stained with 1% AB (pH 2.5) for 45 minutes, followed by treatment with 0.5% periodic acid for 10 minutes, and then incubated with Schiff’s reagent for 15 minutes. Nuclei were counterstained with modified Mayer’s hematoxylin for 2 minutes. For IHC, heat-induced epitope retrieval was performed in a steamer (Black & Decker) for 40 minutes with 10 mM citrate buffer solution (pH, 6) or 10 mM Tris-EDTA solution (pH, 8.0) unmasking solution, followed by 20 minutes cooling at room temperature. Endogenous peroxidase activity was quenched with 3% H_2_O_2_ aqueous solution for 10 minutes. For murine tissue sections, antibody non-specific interactions were blocked for 30 minutes with normal goat, rabbit, or swine serum (Vector Laboratories, 1:5) in Antibody Diluent OP Quanto (Thermo Fisher Scientific), depending on the species of the secondary antibodies. Slides were incubated with primary antibody overnight at 4°C (Table 3). Tissues were then incubated with the corresponding biotinylated secondary antibody for 30 minutes, followed by signal amplification with an avidin/biotin detection system (Vectastain ABC kit, Vector Laboratories) for 30 minutes. For human tissues, the REAL EnVision Detection System, Peroxidase/DAB (Dako) was used after primary antibody incubation. Diaminobenzidine (DAB) was used as chromogenic substrate, with an incubation time of 5 minutes. Nuclear contrast was performed with modified Mayer’s hematoxylin for 1 minute. All slides were dehydrated, clarified, and permanently mounted with a xylene-based mounting medium. For multiplex IHC, a Double Opal multiplex staining (Akoya Biosciences) was performed using the Ventana Discovery Ultra automated platform (Roche). After tissue initial pretreatment as previously described, sequential staining was performed using ImmPress HRP polymers (Vector Laboratories) in combination with Opal-tyramide signal amplification (TSA). Anti-β-catenin antibody (1:50 dilution) was first incubated for 1 hour at 37 °C. Detection was achieved using ImmPress goat anti-mouse horseradish peroxidase (HRP) polymer, incubated for 32 minutes, followed by fluorophore development with Opal 690. An HRP inactivation/stripping step was performed before incubation with the anti-PPARγ antibody (1:50 dilution) for 8 hours at room temperature. Detection was carried out using ImmPress goat anti-rabbit HRP polymer, incubated for 32 minutes, followed by signal development with Opal 570. Slides were counterstained with 4′,6-diamidino-2-phenylindole (DAPI) and mounted using an anti-fade medium (Thermo Fisher Scientific). Whole-slide fluorescence imaging was performed using the PhenoImager HT system (Akoya Biosciences) running software version HT 2.0, with multispectral image acquisition and spectral unmixing capabilities. Images were analyzed using the QuPath (v0.6.0) software.

**Table 3:** List of Antibodies Used in This Study

Antigen	Species	Dilution (assay)	Reference	RRID	Source
ACTB	Mouse (Clone 4)	1:2000 (WB)	sc-47778	AB_626632	Santa Cruz Biotechnology
β-Catenin	Mouse (Clone 14)	1:100 or 1:1000 (IHC)^a^	610153	AB_397554	BD Biosciences
GAPDH	Mouse (Clone 0411)	1:1000 (WB)	sc-47724	AB_627678	Santa Cruz Biotechnology
KRT20	Rabbit (Clone D9Z1Z)	1:750 (IHC)	#13063	AB_2798106	Cell Signalling Technology
KI67	Rabbit (Clone SP6)	1:1000 or 1:2000 (IHC)^a^	ab16667	AB_302459	Abcam
MEX3A	Rabbit	1:500 (IHC)	ab79046	AB_2266271	Abcam
MEX3A	Rabbit	1:2000 (WB)	PRS4869	AB_1853839	Merck
PPARγ	Rabbit (Clone C26H12)	1:300 or 1:800 (IHC)^a^; 1:1000 (WB)	#2435	AB_2166051	Cell Signalling Technology
VIL1	Mouse (Clone 1D2C3)	1:200 (IHC); 1:1000 (WB)	sc-58897	AB_2304475	Santa Cruz Biotechnology
Anti-rabbit (Biotinylated)	Swine	1:100 (IHC)	E0353	AB_2737292	Dako
Anti-mouse (Biotinylated)	Rabbit	1:100 (IHC)	E0354	AB_2687571	Dako
Anti-mouse (HRP conjugated)	Goat	1:2000 (WB)	sc-2005	AB_631736	Santa Cruz Biotechnology
Anti-rabbit (HRP conjugated)	Goat	1:10000 (WB)	#7074	AB_2099233	Cell Signalling Technology

GAPDH, glyceraldehyde 3-phosphate dehydrogenase; HRP, horseradish peroxidase; IHC, immunohistochemistry; MEX3A, Mex-3 RNA binding family member A; PPARγ, peroxisome proliferator-activated receptor gamma; WB, Western blot.

a Mouse tissues or human/mouse organoids, respectively.

### RNA ISH

The single-plex RNAscope 2.5 HD-RED ISH assay (Bio-Techne) was performed in FFPE tissue sections according to the manufacturer’s instructions, with the following modifications: (1) after deparaffinization and rehydration, sections were subjected to the mild pre-treatment protocol; (2) AMP5 incubation was performed for 40 and 45 minutes for human *LGR5* (Hs-LGR5, ref. 311021) and mouse *Lgr5* (Mm-Lgr5, ref. 312171), respectively, and for 1 hour for mouse *Mex3a* (Mm-Mex3a-E2-CDS, ref. 318551); and (3) sections were counterstained with fast green stain solution (Thermo Fisher Scientific) for 1 minute. Slides were dried for 15 minutes at 60°C, clarified and permanently mounted with Vectamount (Vector Laboratories). Incubations at 40°C were performed in a HybEZ hybridization oven (Advanced Cell Diagnostics). Quantification of mRNA was performed using the trainable Weka segmentation classifier, according to recommendations of a Technical Note (TS 46-003/Rev B/Date 04112019) from Advanced Cell Diagnostics.

### Conditioned Media Production for Organoid Cultures

Conditioned media (CM) for organoid cultures was produced using human embryonic kidney (HEK)293T cells stably transfected with a HA-mouse R-spondin1 (RSPO1)-Fc vector (a kind gift from Calvin Kuo, Stanford University), HEK293T cells stably transfected with a mouse NOG-Fc vector (a kind gift from Gijs van den Brink and Vanesa Muncan, Amsterdam University Medical Centers), and murine L cells stably transfected with a WNT3A expressing vector (ATCC CRL2647). All cell lines were routinely cultured in T75 flasks with Dulbecco’s Modified Eagle Medium (DMEM; Thermo Fisher Scientific) + 10% (v/v) fetal bovine serum (FBS; Biowest) with appropriate selection antibiotics. For CM production, these were split (1:5) into 4 T75 flasks with complete DMEM without selection antibiotics. When reaching confluence, cells from 2 T75 flasks were combined into one T175 culture flask with Advanced DMEM/F12, 10% (v/v) FBS, 10 mM HEPES and 1× GlutaMAX (both Thermo Fisher Scientific). After 7 days, CM was harvested, filtered with a 0.22-μm PES filter and stored at −20°C until use.

### Mouse-Derived CRC Tumoroids Culture

Focal lesions were isolated from the distal colon and cut into small pieces of around 5 mm^2^. The epithelial tissue was enzymatically separated from the underlying mesenchymal compartment by incubation with ACCUMAX cell detachment solution plus 10 μM Y-27632 ROCK inhibitor (both from StemCell Technologies) for 30 minutes at 37°C with agitation. The cell suspension was centrifuged and single cells embedded in Matrigel growth factor reduced basement membrane matrix (Corning). The cell/matrix suspension was plated in each well of a pre-warmed 24-well plate and incubated for 30 minutes at 37°C, 5% CO_2_. After complete polymerization, 500 μL of murine tumoroid medium was added to each well, composed of Advanced DMEM/F12, 10 mM HEPES, 1× GlutaMAX, 1× N-2 Supplement, 1× B-27 Supplement (all Thermo Fisher Scientific), 100 μg/mL primocin (InvivoGen), and 10% (v/v) NOG-CM, supplemented with 10 μM Y-27632 (StemCell Technologies) only during establishment and passaging. Culture media was replaced every 2 days and tumoroids passaged every week. Assays were conducted after culture stabilization (at least 3 passages). Tumoroids size, morphology, and markers expression were assessed after 7 days of culture. For PPARγ pathway activation, tumoroids were treated with 50 μM ROSI (Tocris Bioscience) or vehicle (DMSO) for 5 days.

### Patient-Derived CRC Tumoroids Culture

Tumor and matched normal-like tissues obtained from patients with CRC undergoing surgery were enzymatically processed with a Collagenase Type XI (Merck), DNAse I and Dispase cocktail solution (both from StemCell Technologies), plus 10 μM Y-27632 for 30 minutes at 37°C under agitation. The cell suspension was centrifuged and single cells embedded in Matrigel as indicated above. Normal colon organoids were maintained in human colon organoid media composed of Advanced DMEM/F12, 10 mM HEPES, 1× GlutaMAX, 1× N-2 Supplement, 1× B-27 Supplement, 1.25 mM N-acetylcysteine (StemCell Technologies), 10 nM gastrin I (Merck), 5 μM SB202190 (Merck), 500 nM A83-01 (Tocris Bioscience), 50% (v/v) WNT3A-CM, 10% (v/v) NOG-CM, 10% (v/v) RSPO1-CM, 50 ng/mL EGF (Peprotech), 100 μg/mL primocin, 1 μM CHIR99021 (StemCell Technologies) and 10 μM Y-27632 (only during establishment and passaging). CRC-derived tumoroids were kept in human CRC tumoroid media, which is similar to human colon organoid media but without the addition of WNT3A-CM and CHIR99021. Culture media was replaced every 2 days and tumoroids passaged every 2 weeks. Assays were conducted after culture stabilization (at least 3 passages). Tumoroids size, morphology, and markers expression were assessed after 14 days of culture. To address chemosensitivity, tumoroids were plated in 96-well culture plates and treated with 5-FU (Merck) plus oxaliplatin (MedChemExpress) in a 25:1 ratio equivalent to the combined IC50 dose (282.5 μM 5-FU and 11.3 μM oxaliplatin) or vehicle-treated for 72 hours. Cell viability was measured using the CellTiter-Glo 3D Cell Viability assay (Promega) following the manufacturer’s instructions.

### Establishment of CRISPR/Cas9-Mediated MEX3A KO Tumoroid Lines

A single-guide RNA (sgRNA) targeting the MEX3A coding region (https://genome.ucsc.edu/cgi-bin/hgTracks) was cloned into a SpCas9-EGFP vector (PX458; Addgene #48138) using a previously described protocol (Table 2).^56^ The patient-derived CRC tumoroid line 005T was dissociated and plated in collagen type I (ibidi) coated plates. Adherent cells were transfected with 4 μg of the PX458-sgMEX3A expressing vector using lipofectamine 2000 reagent (Thermo Fisher Scientific). Cells were enzymatically removed from the collagen 48 hours after transient transfection, single EGFP+ cells collected by FACS using a BD FACS Aria II sorter (BD Biosciences) and plated in collagen type I-coated 96-well plates to establish monoclonal populations of putatively edited tumor cells. Downstream analysis was performed after reestablishment of the culture in Matrigel. KO efficiency of different clones was evaluated by Western blot analysis of MEX3A protein expression and by DNA Sanger sequencing.

### Protein Extraction and Western Blot

Cells were lysed on ice for 30 minutes with lysis buffer containing 20mM Tris-HCl (pH, 7.5), 150 mM NaCl, 2 mM EDTA, 0.1% (v/v) sodium deoxycholate, and 0.1% (v/v) sodium dodecyl sulfate supplemented with 1× Complete protease inhibitor cocktail (Roche Applied Science), 20 mM NaF, 1 mM PMSF, and 1mM Na_3_VO_4_. Lysates were centrifuged for 15 minutes at 17,000 × *g* at 4°C and the supernatant recovered. Protein concentration was determined with BCA Protein Assay kit (Thermo Fisher Scientific). Twenty to 50 μg of each protein extract were run in a 10% sodium dodecyl sulfate-polyacrylamide gel electrophoresis (SDS-PAGE) gel, transferred to a nitrocellulose membrane, and incubated overnight with the desired antibodies (Table 3) after which signals were revealed with ECL detection kit (GE Healthcare Life Sciences). Actin or glyceraldehyde 3-phosphate dehydrogenase (GAPDH) levels were used to normalize protein expression, and quantification was performed using the Fiji software.^57^

### Site-Directed Mutagenesis and HyperTRIBE Constructs

A previously published MEX3A expression vector^28^ was used together with a *Drosophila melanogaster* ADARcd expression vector (a kind gift from Jean-Pierre Rouault, Centre de Recherche en Cancérologie de Lyon). An inactive MEX3A RNA-binding mutant (MEX3AΔKH) was generated by introducing a double amino acid change in both KH domains: R150D and Q151D in KH1, P241D and K242D in KH2, previously predicted to impair nucleic acid binding without causing significant structural changes or compromising domain stability.^26^ The hyperactive version of *Drosophila’s* ADARcd was generated by introducing E458Q, the human ADAR2 E488Q matching mutation, previously described to increase human’s ADAR2cd editing efficiency and reduce sequence recognition bias.^58^^,^^59^ All mutations were introduced with the Q5 Site-Directed Mutagenesis Kit (New England Biolabs) using oligonucleotides containing the desired mutations (Table 2). The ADARcd, MEX3A, and MEX3AΔKH coding sequences were amplified with overlapping primers (Table 2), allowing assembly of adjacent fragments. Amplified fragments were cloned into the pCW57-RFP-P2A-MCS lentiviral vector (Addgene #78933) after *BamH*I/*Pst*I digestion, using the NEBuilder High-Fidelity DNA Assembly Cloning Kit (New England Biolabs), generating MEX3A-ADARcd and MEX3AΔKH-ADARcd HyperTRIBE vectors. Sanger sequencing was performed to confirm proper cloning using the BigDye Terminator (v3.1) Cycle Sequencing Kit (Thermo Fisher Scientific). Sequencing products were purified with Sephadex G-50 Fine DNA Grade (GE Healthcare) before analysis.

### Lentiviral Particles Production and SW480 Cell Transduction

HEK293T (ATCC CRL-3216) cell line was used for production of replication-incompetent lentiviral particles to deliver the HyperTRIBE vectors. Briefly, 2 μg psPAX2 packaging vector (Addgene #12260), 2 μg pCMV-VSV-G envelope vector (Addgene #14888), and 4 μg MEX3A-ADARcd or MEX3AΔKH-ADARcd vectors were diluted in Opti-MEM (Thermo Fisher Scientific) and transiently transfected into HEK293T cells using lipofectamine 2000 reagent. Viral particle-containing supernatants were obtained 48 hours later, filtered through a 0.45-μm PES filter, and precipitated with PEG-it Virus precipitation kit (System Biosciences). The human colorectal carcinoma cell line SW480 (ATCC CCL-228) was plated and left for 24 hours in DMEM + 10% (v/v) FBS to adhere. The media was then replaced by fresh media containing diluted viral supernatant plus 15 μg/mL polybrene (Merck). After incubation for 96 hours, the media was removed and selection of transduced cells initiated with 10 μg/mL puromycin (InvivoGen). SW480 HyperTRIBE stable cell lines were plated for system induction with 1 μg/mL DOX. After 48 hours of treatment, TurboRFP+ cells were collected by FACS, along with noninduced cells, pelleted and stored at −80°C for downstream analysis.

### RNA Extraction and Reverse Transcription Quantitative Polymerase Chain Reaction

Total RNA was extracted using TRI Reagent according to the manufacturer’s instructions (Merck). RNA was quantified using a NanoDrop 1000 spectrophotometer (Thermo Fisher Scientific) and 2 μg of total RNA reverse-transcribed using the NZY Reverse Transcriptase (NZYTech). Analysis of mRNA expression was performed by quantitative real-time polymerase chain reaction (RT-qPCR) in an ABI prism 7500 system with PowerUp SYBR Green Master Mix (Thermo Fisher Scientific) and specific primer pairs (Table 2). Each sample was quantified in triplicate, and specificity was confirmed by dissociation analysis. Gene expression was calculated through the relative standard curve method, with *18S* rRNA levels used for target gene abundance normalization.

### RNA-Seq

SW480 HyperTRIBE total RNA quantification and quality control were assessed using a 2100 Bioanalyzer instrument (Agilent Technologies). Only samples with an RNA integrity number (RIN) above 9 were considered for further analysis. RNA samples were processed for rRNA removal using the Ribo-Zero kit (Illumina). Then, mRNA was randomly fragmented by adding fragmentation buffer and cDNA synthesized using random hexamers, after which a custom second-strand synthesis step was performed. This was followed by purification with AMPure XP beads, terminal repair, polyadenylation, sequencing adapter ligation, size selection, and degradation of second-strand U-contained cDNA by the USER enzyme. After the final PCR enrichment, a strand-specific cDNA library was generated using the NEBNext Ultra II Directional RNA Library Prep kit for Illumina (New England Biolabs). These procedures were performed by Novogene.

### Bioinformatic Analysis of RNA-Seq Data

Trimming of the reads was first carried out using Trimmomatic (v0.39), with the following non-default settings recommended for the HyperTRIBE pipeline (https://github.com/rosbashlab/HyperTRIBE): Head-Crop: 6; Number of leading bases for trimming: 25; Number of trailing bases for trimming: 25; Average quality of 3 consecutive bases: 25; Minimum length 19; Adapters trimming (using TruSeq3 adapters): TruSeq3-PE.fa:2:30:10:2:keepBothReads. The human reference genome, build GRCh38 (patch release 12), and the curated gene structure were retrieved from the UCSC Genome Browser. The alignment of the paired-end RNA-seq reads to the human genome assembly was performed using STAR aligner (v2.7.8a).^60^ Besides default options, the following were applied specifically for HyperTRIBE: outFilterMismatchNoverLmax: .07; outFilterMatchNmin: 16; outFilterMultimapNmax: 1. The aligned reads were outputted in BAM format sorted by coordinates. Aligned reads were further filtered in samtools (v1.12), considering only alignments with a mapping Phred quality score above 10. Duplicate marking of reads was carried out in MarkDuplicates (Picard) tool using the “REMOVE_DUPLICATES” option. Next, we followed the GATK workflow^61^ for variant calling in RNA-seq to identify mutations in the aligned reads. Prior to the variant calling step, as per the GATK recommendations, a base quality recalibration step was carried out in order to detect and fix systematic errors on the quality scores. We then restricted the analysis to mutations within annotated RNA transcripts, retrieved from the National Center for Biotechnology Information (NCBI) RNA reference sequence (RefSeq) collection, calling out A-to-G mutations in transcripts encoded by the forward strand and T-to-C mutations in transcripts encoded by the reverse strand. The filtered sets of RNA editing events from RNA-seq libraries of the same experimental condition were combined, and the number of reads containing the reference (A or T) and alternative (G or C) alleles from each library at each site were counted. We applied a beta-binomial distribution to model RNA edit frequencies,^62^ comparing the MEX3A-ADARcd induced condition against both the MEX3AΔKH-ADARcd induced and the NIC samples. The *P* values from all sites were adjusted to control for FDR using a Benjamin-Hochberg correction. Significant sites were determined by filtering for FDR-adjusted <.001, an expression level of at least 1 FPKM, and a minimum of 20 reads counts in each MEX3A-ADARcd replicate. High confident target genes were retained considering no editing in controls (G or C = 0) and a differential edit frequency of at least 25%. For de novo motif discovery, we extracted the sequences extending 500 bp from both sides of each edit site in the 3′UTR regions of the previously identified transcripts and considered these windows as the target sequence pool for the HOMER software. Overlapping sequences were merged into a single sequence, and background sequences of similar length were randomly selected from 3′UTRs in the genome that did not overlap with the target sequence. The search was limited to enriched motifs of 6, 7, or 8 nucleotides in length, and a regional oligomer autonormalization of up to 3 nucleotides in length. For differential gene expression analysis, the aligned reads previously mapped were counted using HTSeq-Count.^63^ Differential expression analysis between paired tests were identified by the DESeq2 package, which applies a negative binomial distribution test to model gene counts and test for differential expression. Only genes with a *P* value threshold adjusted for multiple testing (Benjamini-Hochberg method) < .01 were considered.

### Statistical Analyses

At least 3 biological replicates were done for each experiment, with 3 replicates per assay. Statistical analysis of data was performed using the GraphPad Prism software, and differences between groups were considered statistically significant at a *P* < .05. For statistical analysis of differences between tumor burden in mice, the χ^2^ test was applied, whereas differences in tumoroid morphology were accessed through Fisher’s exact test. Differences for marker expression level, tumor area, tumoroid diameter, wall thickness, and response to chemotherapy were determined using unpaired Student’s *t*-test or Mann-Whitney test for data not following normal distribution. Differences in RT-qPCR analysis and number of editing events in HyperTRIBE were evaluated with 1-way analysis of variance (ANOVA). For human CRC tissues, results are expressed as a percentage or mean ± standard deviation (SD). For analysis of the relationship between patient’s age and biomarker expression, we used 2-tailed unpaired Student’s *t*-test. Pearson’s χ^2^ test was used in the statistical analysis of parameters where all classes had an expected count above 10, otherwise, Fisher’s exact test was implemented.
